# Arbuscular Mycorrhizal Fungal Networks Vary throughout the Growing Season and between Successional Stages

**DOI:** 10.1371/journal.pone.0083241

**Published:** 2013-12-17

**Authors:** Alison Elizabeth Bennett, Tim John Daniell, Maarja Öpik, John Davison, Mari Moora, Martin Zobel, Marc-André Selosse, Darren Evans

**Affiliations:** 1 Ecological Sciences, The James Hutton Institute, Dundee United Kingdom; 2 Department of Botany, Institute of Ecology and Earth Sciences, University of Tartu, Tartu, Estonia; 3 Muséum national d'Histoire naturelle (Département Systématique et Evolution), Paris, France; 4 School of Biological, Biomedical and Environmental Sciences, University of Hull, Kingston upon Hull, United Kingdom; University Copenhagen, Denmark

## Abstract

To date, few analyses of mutualistic networks have investigated successional or seasonal dynamics. Combining interaction data from multiple time points likely creates an inaccurate picture of the structure of networks (because these networks are aggregated across time), which may negatively influence their application in ecosystem assessments and conservation. Using a replicated bipartite mutualistic network of arbuscular mycorrhizal (AM) fungal-plant associations, detected using large sample numbers of plants and AM fungi identified through molecular techniques, we test whether the properties of the network are temporally dynamic either between different successional stages or within the growing season. These questions have never been directly tested in the AM fungal-plant mutualism or the vast majority of other mutualisms. We demonstrate the following results: First, our examination of two different successional stages (young and old forest) demonstrated that succession increases the proportion of specialists within the community and decreases the number of interactions. Second, AM fungal-plant mutualism structure changed throughout the growing season as the number of links between partners increased. Third, we observed shifts in associations between AM fungal and plant species throughout the growing season, potentially reflecting changes in biotic and abiotic conditions. Thus, this analysis opens up two entirely new areas of research: 1) identifying what influences changes in plant-AM fungal associations in these networks, and 2) what aspects of temporal variation and succession are of general importance in structuring bipartite networks and plant-AM fungal communities.

## Introduction

The analysis of bipartite mutualistic networks is a powerful tool for understanding the structure and dynamics of mutualistic interactions with multiple partners. Analysis of networks can determine the proportion of specialists and generalists within a focal community, the interactions between seemingly unrelated species, system complexity and functioning, as well as identify the organisms most strongly influencing these community properties [1]. As a result, bipartite network analysis of mutualisms is a growing area of interest in a wide variety of fields and study systems, though the focus has predominantly been on interactions between plants and animals [2].

Despite the power of network analyses to examine community structure, these analyses have rarely been applied to experimental or seasonal temporal data (but see [3], [4]–[7]). Until recently, network analyses often combined data on the same communities from multiple years [8], [9], and seasons [9] to build a single network that assumed no differences between years and seasons. Temporal dynamics such as succession and seasonal variation are fundamental ecological processes, and understanding the influence of these processes on networks will help reveal the basic properties that structure communities. As a result, the incorporation of successional and seasonal dynamics in network analyses is crucial.

Few studies have focused on temporal dynamics in networks between months and seasons and those that have focused on non-bipartite aquatic [4], [5] and bi-partite plant-pollinator systems [7], [10], [11]. These latter studies have revealed strong shifts in network structure, particularly connectance (the proportion of realized links between species in the network [12]), throughout the year, and increases in the number of links. This further supports the assertion that lumping interaction data from multiple time points in a season likely biases the analysis of bipartite networks [11] concealing aspects of network structure which could have strong implications for the application of networks in ecosystem assessments and conservation.

The small number of studies examining seasonal dynamics in terrestrial systems is likely due to the time required to gain sufficient observations to produce a highly resolved network model. However, the prevalence of new rapid screening molecular techniques (such as high sample throughput techniques (e.g. T-RFLP [13]), and cloning and sequencing [14],[15], and next generation sequencing [4], [15], [16]) is rapidly improving our ability to gather data in a timely and cost-effective fashion. These technologies allow researchers to sample at distinct time points with sufficient resolution for the creation of replicated temporal quantitative networks in systems (such as arbuscular mycorrhizal (AM) fungal-plant networks) that have rarely been studied in detail before.

The number of studies examining how networks differ between successional stages is even smaller than the number examining seasonal dynamics. We know of only two studies examining successional dynamics: an ant-plant network that was re-examined at the same site ten years after the initial analysis [6], and a study of plant-pollinator networks along a 130 year chronosequence of glacier development [3]. Both studies found a decrease in the proportion of specialists with time due to increases in the numbers of partners within the network [3], [6], but found opposite patterns for link density (mean number of interactions per species [1]) and connectance [12]. In the ant-plant system both connectance and linkage density increased with time [6], and the authors argued that these changes were due not to increases in network size but instead increases in the interactions between particular members of the original community that grew over time (due to their invasive nature). By contrast, in the plant-pollinator system linkage density remained constant with time but connectance tended to decrease [3], and the authors argued that this was due to an overall increase in network size. Both of these studies examined differences between successional stages but combined one [3] or two years [6] of data to make comparisons thereby ignoring seasonal differences within their sites. Thus, no previous netowrk anlayses have incorporated both seasonal and successional dynamics, and the few existing analyses of successional networks may have been biased by the lumping of seasonal data [10], [11].

Network analysis stands to provide a great wealth of information about belowground mutualistic organisms such as AM fungi where direct observations have been greatly limited [17]. Rapid screening molecular techniques are now being used to assess changes in temporal dynamics between successional stages in AM fungal-plant interactions [13]–[16], [18]. The AM fungal-plant mutualism is arguably the most important free-living mutualism on the planet—these fungi appear to have facilitated plant colonization of land in the early Devonian [reviewed in 19], associate with more than 80% of all plant species [17], and contribute to plant diversity in natural systems (reviewed in [20]). AM fungi act as a secondary root system that aids in nutrient (predominantly phosphorus, some nitrogen, and trace minerals) uptake and improved water availability for host plants, and in return the fungi gain carbon from their host plants [17]. The effect of biotic (e.g. soil pathogens [21]) and abiotic (including nutrient availability, pH, and light availability [15], [22]) factors on plant growth is also mediated by interactions with AM fungi.Using molecular techniques, researchers have shown that plants associate with multiple AM fungal species simultaneously, although some combinations of plant and fungal partners occur more frequently than others [14], [23]. Seasonal temporal variation occurs in the AM fungal-plant mutualism [15], [24], and anthropogenic disturbance can strongly influence the abundance, diversity, and community composition of AM fungi in a system [25], [26]. AM fungal spore community composition has also been shown to shift between successional stages [27], [28] which may contribute to variation in plant-AM fungal associations [13], [14], [16]. As a result, both seasonal and successional dynamics influence AM fungal and plant communities.

Recent analyses of AM fungal-plant networks have revealed that, like most bipartite mutualistic networks, they are nested (defined by Bascompte & Jordano [2] as “a pattern of interaction in which specialists interact with species that form perfect subsets of the species with which generalists interact.”) [29], [30], and, potentially depending on the number of AM fungal genera present in a system, may be modular (consisting of sub-groups of organisms more likely to interact within the sub-group than with organisms outside the sub-group) [29].

However, as with most mutualistic bipartite networks, we know little about how seasonal and successional dynamics within the plant-AM fungal mutualism alter network structure, and, when considering all bipartite mutualistic networks, we do not know if seasonal and successional dynamics interact to influence network structure. As a result, we set out to answer the following questions:

First, does successional stage alter network properties? We predict, as suggested above, that in the absence of invasive species and in the older successional stage there should be more species and therefore higher connectance and linkage density. We also predict, as seen in the ant-plant [6] and plant-pollinator system [3] that specialisation will decrease with time since disturbance because network size will increase with time since disturbance.
Second, do temporal dynamics within a growing season alter network properties? We predict, based on the previous research described above, that links per species and connectance will increase throughout the growing season. This change will likely be due to an increase in the number of links through time because of an increase in the number of organisms in the system between spring and fall. These changes should then result in an increase in AM fungal generality [the effective number of plants per AM fungi (1)] and hence a reduction in plant vulnerability [the effective number of AM fungi per plant (1)] within the network.
Finally, do successional stage and seasonal dynamics interact to influence network properties? This last question is important for determining whether successional analyses of bipartite network structure are likely to be biased if temporal dynamics are not included. Many of our predictions concerning the effects of successional and temporal dynamics on network properties are similar (e.g. connectance), so we predict these effects will likely be magnified when seasonal and successional changes coincide.

We addressed these questions in a replicated well characterized AM fungal-plant system in the Estonian boreonemoral forest ecosystem [13], [14], [16], [18], [31]–[35].

## Materials and Methods

**Figure 1:**
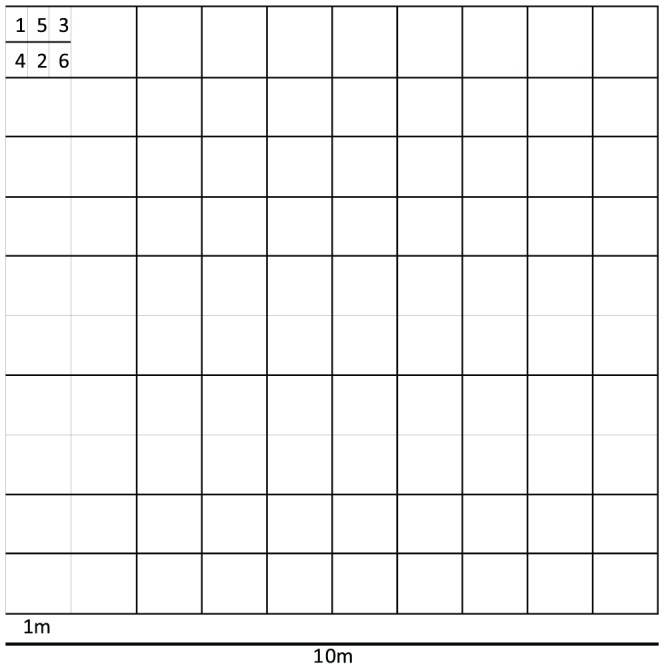
The “zig-zagged” sub-subsampling scheme within each 1 m×1 m subplot within each 10 m×10 m plot. The numbers labelling each sub-subplot correspond to a sampling time (1–6; only time points 1 (early June), 2 (late July), and 3 (early October) were used in this analysis). One individual of each of the 11 plant species was removed from the appropriate sub-subplot in each 1 m×1 m subplot at each time point for a total of 100 individuals of each species removed from each plot at each sampling time (when possible).

This study re-analyzes data from Davison et al. [14] and Öpik et al. [18], and therefore plant and AM fungal identification details are described in Davison et al. [14]. Briefly, three separate 10×10 m forest plots in the Koeru forest area in central Estonia were sampled within two different successional stages: three old growth spruce forest (old successional stage), and three spruce forest clearcut and planted 25 years prior to sampling (young successional stage) for a total of 6 plots sampled at three times during the growing season. Plant roots were sampled from each plot in early June, late July, and early October 2003 (see sampling diagram, Figure 1) for molecular analysis of the AM fungal communities colonising roots. Plants were identified prior to collection of roots, and samples belonging to 11 of the more common plant species (*Fragaria vesca*, *Galeobdolon luteum* (synonym for *Lamiastrum galeobdolon*), *Hepatica nobilis*, *Oxalis acetosella*, *Trifolium pratense*, *Geranium pratense*, *Geum rivale*, *Hypericum maculatum*, *Paris quadrifolia*, *Veronica chamaedrys*, and *Viola mirabilis*) were collected in each plot at each time point where possible. Not all plant species were present at each sampling time in each plot, and *T. pratense* occurred only in the young successional stage plots while *G. luteum* only occurred in the old successional stage plots. These sampled species were spread among ten different plant families at the study sites, and included 55% of the total understorey plant cover of the sampled plots [31]. Details of the molecular analysis can be found in Öpik et al. [18] and Davison et al. [14]. Briefly, the AM fungal nuclear SSU rRNA gene was targeted using the primers NS31 and AM1 (as described in [18]), and resulting PCR products were cloned. Forty-eight of the resulting colonies per individual plant were picked and stored, and 16–32 of these colonies were used for plasmid isolation and Sanger sequencing. Single strand sequences were generated and compared using TOPALi [36] along with known taxa from the Maarj*AM* database of Glomeromycota sequences [37] on a neighbour joining phylogenetic tree. Sequences were grouped into phylogroups based on the tree at ≥97% sequence similarity. In the subsequent analyses, the respective virtual taxon (VT) nomenclature of the Maarj*AM* database of Glomeromycota sequences is used. VT are phylogroups which are defined on the basis of bootstrap support and sequence similarity ≥97% across data originating in individual case studies [37], and used as a proxy for species throughout the remainder of this paper.

To determine how well our sampling method estimated the diversity of AM fungal species in each host plant, successional stage, and sampling time we produced rarefaction curves using the ‘exact’ method within the specacum() function [38] uisng the package ‘vegan’ in R 2.15.3 (R Development Core Team, 2013)[39]. Using the specpool() function in ‘vegan’ we also calculated the Chao total richness estimates for each group (plant species, successional stage, or time point) [40].

Plant and fungal associations were examined in replicate matrices constructed for each combination of site, successional stage and sampling time (18 matrices in total). Network structures were calculated using the package ‘bipartite’ in R. There is an array of potential network descriptors available in the literature [41], [42]. To address our hypotheses we examined both qualitative metrics (that examine presence/absence data) and quantitative metrics (which take into account the frequency of interaction) following Tylianakis *et al*. [1]. We analysed the qualitative metrics network specialization (ranges between 0 (no specialisation) and 1 (complete specialisation)) [referred to as H2'; 43], network connectance [the proportion of realized links, C; 12], links per species (sum of links divided by number of species) and the quantitative metrics linkage density (mean number of interactions per species), generality (the effective mean number of plants associating with single AM fungus), and vulnerability [1] to determine whether successional stage influenced the interactions between AM fungi and plants. We used Generalised Linear Models to examine the effects of successional stage and sampling time on each network descriptor following Tylianakis *et al.* [1]. H2' data was arcsin square root transformed prior to analysis.

By observing the networks we could discern changes in species associations throughout the growing season. To determine whether seasonal or successional dynamics influenced these changes we used the metric turnover rate (t) [44]. This metric has previously been used to determine the consistency in network structure between years, but here we adapt it to examine the difference in network structure between sampling times within each successional stage. Turnover rate is calculated for each partner group (plants and AM fungi) as well as the links between species.

Finally, we calculated the number of unique interactions for old and young successional stage networks by pooling data and comparing ‘robustness’ [45] to species extinction. In our analysis of ‘robustness’ plants were randomly eliminated from the population, and the number of secondary extinctions measured. ‘Robustness’ varies between 0 and 1, and networks with a robustness closer to 1 experienced fewer secondary extinctions.

## Results

**Figure 2:**
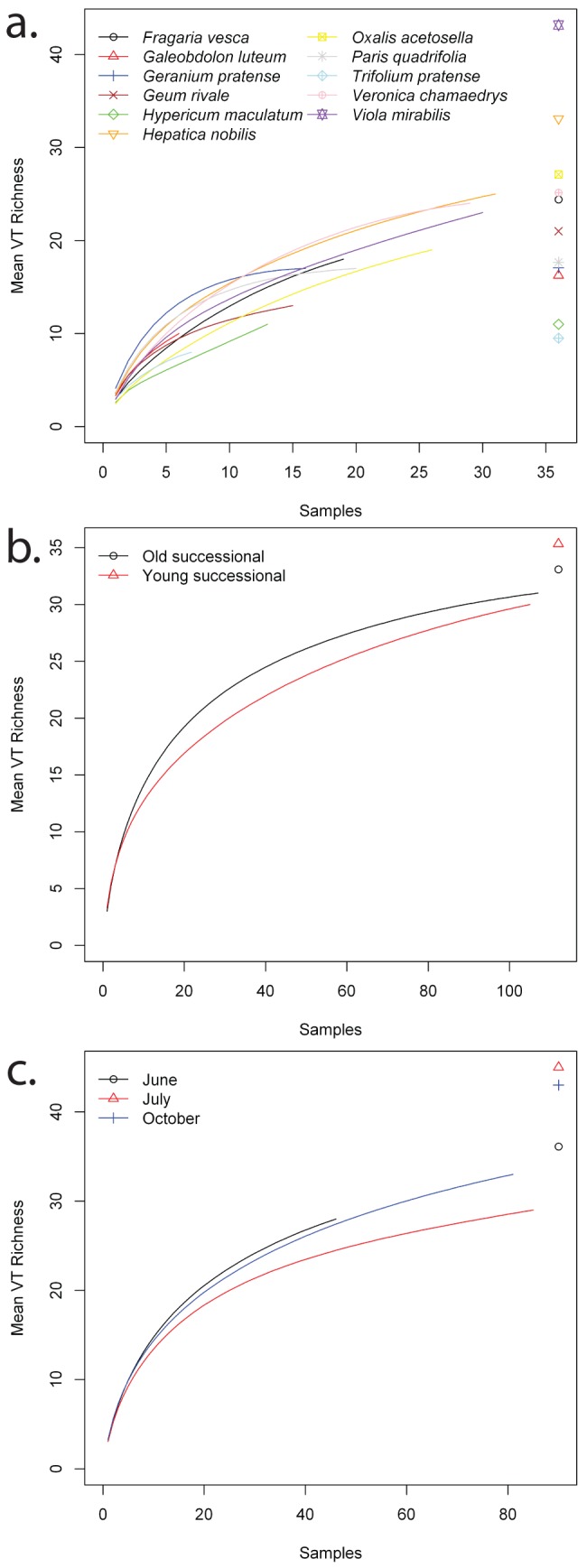
Rarefaction curves for each of the a) plant species, b) successional stages, and c) sampling times. Chao estimates of total richness are included on the right side of each graph.

**Figure 3:**
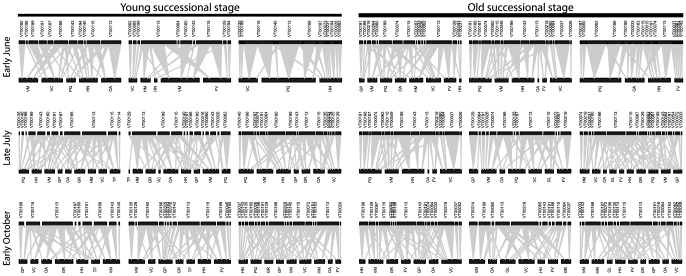
Bipartite Networks from the three plots within each successional stage (Young Successional Stage networks are located in the first three columns, Old Successional Stage networks are located in the last three columns) by sampling date (early June, late July, and early October) (represented by rows). AM fungal “virtual taxa” (VT) (used as a proxy for species) are numbered and are represented at the top of each network. Plant species, located across the bottom of each network, are represented by two letter abbreviations for each plant species. The abbreviations are as follows: FV =  *Fragaria vesca*, GL =  *Galeobdolon luteum*, HN =  *Hepatica nobilis*, OA =  *Oxalis acetosella*, TP =  *Trifolium pratense*, GP =  *Geranium pratense*, GR =  *Geum rivale*, HM =  *Hypericum maculatum*, PQ =  *Paris quadrifolia*, VC =  *Veronica chamaedrys*, and VM =  *Viola mirabilis*).

**Figure 4:**
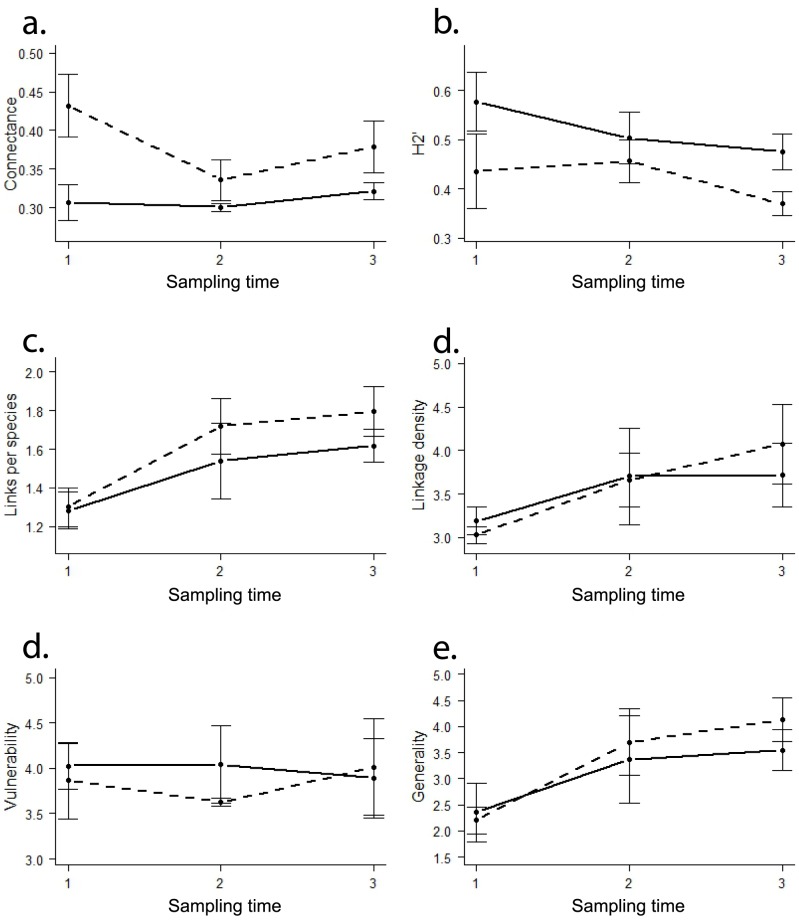
Graphs of the mean and standard error of a) Connectance, b) network specialization (H2'), c) Links per species, d) Linkage density, e) Vulnerability, and f) Generality graphed by successional stage (old (solid lines), young (dashed lines)) across the three sampling times (1 (early June), 2 (late July), and 3 (early October)).

For all successional stages and sampling times as well as the majority of plant species, sample rarefaction curves approached an asymptote and estimated (Chao) total richness did not greatly exceed observed species richness (Table 1; Figure 2). The ratio of observed∶estimated (Chao) richess for the entire dataset was 0.94, and ratios of observed∶estimated richness for sampling times and successional stages were also high (Table 1). This suggests that our sampling effort was relatively effective and that, in most cases, our sampling scheme appropriately estimated the diversity of AM fungal species present in plant roots. Similarly, network descriptors were generally consistent between replicate plots within each successional stage (Figure 3), and tended to exhibit relatively low variation (Figure 4).

**Table 1:** The Chao estimates of total species richness, standard error, and ratio of observed richness to estimated Chao richness for the entire dataset, each successional stage, each sampling time, and each plant species.

Variable	Variable Level	Chao estimate	Chao estimate Standard Error	Observed∶Estimated
All records	none	42.72	2.79	0.94
Successional stage	old	33.08	2.51	0.94
	young	35.33	4.93	0.85
Sampling Time	June	36.10	7.10	0.78
	July	45.00	16.50	0.64
	October	43.00	8.37	0.77
Plant species	FV	24.40	5.92	0.74
	GL	16.25	7.55	0.62
	GP	17.08	0.34	0.10
	GR	21.00	11.66	0.62
	HM	11.00	0.00	1.00
	HN	33.10	7.10	0.76
	OA	27.10	7.10	0.70
	PQ	17.67	1.31	0.96
	TP	9.50	2.29	0.84
	VC	25.14	1.51	0.95
	VM	43.17	17.42	0.53

=  *Fragaria vesca*, GL =  *Galeobdolon luteum*, HN =  *Hepatica nobilis*, OA =  *Oxalis acetosella*, TP =  *Trifolium pratense*, GP =  *Geranium pratense*, GR =  *Geum rivale*, HM =  *Hypericum maculatum*, PQ =  *Paris quadrifolia*, VC =  *Veronica chamaedrys*, and VM =  *Viola mirabilis*). Plant species are represented by two letters, and the abbreviations represent the following plant species: FV

Analysis of the network structures produced for each plot in each sampling time (Figure 3) revealed a significant increase from old to young successional stage in connectance (the proportion of realized links) (*C*: old  = 0.31, young  = 0.38, F_1,12_ = 11.602, p = 0.005; Table 2, Figure 4a) and an increase in specialization (H2': old  = 0.52, young  = 0.42, F_1,12_ = 5.499, p = 0.037, Table 2, Figure 4b). There was also a higher number of AM fungal species per network in the older successional stage (young  = 12.67±0.7 SE, old  = 15.4±0.2 SE), although this difference was not statistically significant. There was no statistically significant effect of successional stage on the number of links per species, linkage density, vulnerability, or generality (Table 2, Figure 4c, d, e, f).

**Table 2:** The results of independent GLMs examining the response variables Connectance, network specialization (H2), links per species, Linkage Density, Vulnerability, and Generality as a function of Successional Stage, Sampling time, and their interactions.

		Connectance		H2		Links/Species		Linkage Density		Vulnerability		Generality	
	df	F	p	F	p	F	p	F	p	F	p	F	p
Succession	1	11.602	**0.005**	5.499	**0.037**	1.387	0.262	0.032	0.861	0.227	0.642	0.330	0.577
Sampling Time	2	1.945	0.185	1.375	0.290	5.713	**0.018**	2.258	0.121	0.062	0.940	4.595	**0.033**
Succession ^*^ Sampling Time	2	1.620	0.238	0.440	0.654	0.261	0.77	0.279	0.761	0.244	0.787	0.237	0.793
Error	12												

the three sampling times (1 (early June), 2 (late July), and 3 (early October)).

By combining plots and comparing all interactions in the successional stages we showed that the number of unique interactions was higher in the old successional stage (old  = 65, young  = 50), and that the old successional stage was also more robust to random plant species extinction (Robustness: old  = 0.62, young  = 0.57).

Temporal dynamics (sampling time) also influenced network structure (Table 2, Figure 4). There was a significant effect of sampling time on the mean number of links per species (June  = 1.29, July  = 1.63, October  = 1.71, F_2,12_ = 5.713, p = 0.018), and generality (or the number of plant species associating with a single AM fungal Virtual Taxon) increased throughout the growing season (F_2,12_ = 4.595, p = 0.033; Table 2, Figure 4c, f). The rate of link turnover (t) decreased for both plants (old  = 0.17, young  = 0.24) and AM fungi (old  = 0.28, young  = 0.49) with time since disturbance, but turnover for AM fungi was always greater than for plants. Turnover rate of links increased with time since disturbance (old  = 0.84, young  = 0.74), and was higher than for both plants and AM fungi.

## Discussion

### Successional stage influenced network structure

In contrast to our prediction specialization was greater in the older than the younger successional stage, and connectance was lower in the older successional stage while there was no effect on linkage density. Robustness was higher in the older successional stage. These results suggest that there were fewer interactions between plant and AM fungal species in the older successional stage, but those interactions were more likely to be specialist interactions. These changes have produced a community with a greater robustness to perturbation (i.e. species extinction) than the community in the younger successional stage.

Very little previous research has examined the successional dynamics of the AM fungal-plant association, and most of that research has focused on the influence of successional dynamics on AM fungal root colonization (without identifying species) or AM fungal spore diversity. In most systems AM fungal spore diversity increases with time but then decreases as forest systems develop [27], although the opposite pattern was observed in the Brazilian tropical dry forest [28]. However, spore diversity is likely not a good predictor of AM fungal diversity in root systems [46]. Prior to the research at the Koeru forest, Estonia, no one had ever examined the actual associations between AM fungi and plants (instead of AM fungal spore diversity or root colonization (without species identity)) throughout succession, so this (and previous research in the Koeru forest system) is the first to document the influence of succession on arbuscular mycorrhizae.

Our results both agree and disagree with previous bipartite network analyses examining successional dynamics in phylogenetically distinct systems. None of our results corresponded with those found in a comparison of ant-plant networks built at the same site in Mexico in 1990 and 2000 [6], but some of our results agreed with an analysis of a plant-pollinator network conducted along a 130 year transect [3]. Whereas no change in link density with successional stage occurred in our network and the plant-pollinator network, there was an increase in link density with time in the ant-plant network. Connectance decreased with time since disturbance in our system and tended to decrease with time in the plant-pollinator system; however, connectance increased with time in the ant-plant system. The authors of the ant-plant study argue that the changes in connectance that occurred in their system were due primarily to changes in the plant community [6] whereas there was little overall change in the plant species sampled between sites in our system which may explain the differences between these two studies. By contrast, where we observed an increase in the proportion of specialists, both the analyses of the ant-plant and plant-pollinator systems observed a relative decrease in the proportion of specialists with time since disturbance. As a result, more network analyses examining these and other properties (e.g. modularity) are required to aid a general understanding of the role of succession in structuring bipartite networks.

It is difficult to determine what differences between the successional stages influenced the AM fungal-plant network structure in our system. Norway spruce, the dominant tree in our study system, is not an AM fungal host, so removal of spruce should not directly impact on AM fungi in this system. However, clear-cutting is a very strong disturbance which imposes major changes in understory environmental conditions and vegetation in this forest system [34]. Moreover, common silvicultural practice in northern Europe (plantation and maintenance of monocultures) produces even-aged coniferous stands [47] altering the structure [33] and reducing the diversity [35] of the herbaceous field layer of predominantly AM fungal plant hosts. These factors produced a significant difference in the structure of the understory plant community in young successional stands compared to unmanaged old growth forest [31], which may in turn have limited the interactions between plant and AM fungal species. As a result, under these management conditions, the time required to restore linkages to their former state is clearly longer than 25 years.

The considerably greater time since a disturbance event in the old growth plots also resulted in higher network specialisation. Network analyses of specialisation do not consider the biological capability of individual species within the network, instead they consider specialisation at the network level. That is, given an expected number of interactions between species within the network, how frequently does the actual number of interactions between species fall below the expected number? Those species with fewer interactions than the expected number increase the degree of specialisation within a network analysis context. Although the other successional bipartite analyses did not produce this result [3], [6], disturbance has been shown in other systems to have a greater negative effect on functional specialists than generalists [48]. Our analysis cannot determine whether the differences in the specialisation within our system are due to the following non-exclusive hypotheses: 1) lack of preferred partners in the early successional stage, 2) loss of rare AM fungal phylotypes with disturbance in the early successional stage, or 3) selection for specialization by plants or fungi in the later successional stage. First, in the young successional stage AM fungi and plants may not be able to associate with preferred partners, and may therefore be forced to associate with a wider group of partners to avoid extinction. Post-disturbance associations may not reflect host preference but rather the local availability of fungal taxa (as AM fungi are dispersal limited and may not easily re-colonize disturbed habitats). If this were to be true, AM fungi that appear as specialists in the older successional stage would appear as generalists in the younger successional stage. Although we cannot definitively test this, it is somewhat supported by two lines of evidence: First, we observed a trend of increased associations with host plants among some AM fungal virtual taxa in the young successional stage (exhibited by Glomus VT00113, VT00115, VT00143, VT00160, VT00166), and, in two cases (AM fungal taxa VT00143 and VT00160), specialist taxa switched to a generalist strategy. Second, the greater number of unique interactions found in the older successional stage also supports the notion that AM fungal specialists in the older successional stage are generalists in the younger stage.

The second hypothesis suggests that rare AM fungal taxa may be lost following disturbance in the early successional stage. Rare AM fungal phylotypes have a greater likelihood of local extinction due to chance in a disturbance event, suggesting that specialists in the old growth forest may be absent in the younger successional stage. The loss of specialist AM fungal host plants could also lead to the loss of specialist AM fungi. Supporting this, an earlier study in the Koeru forest area revealed a distinct set of AM fungi associated only with forest habitat specialist plants—plants which are less likely to be present in the young successional stage [16]. In contrast, our system has previously been shown to be nested [29], [30], and therefore we would expect specialist plants to be associating predomoninantly with generalist AM fungi. Thus the loss of a specialist plant species would then not lead to the loss of a specialist AM fungal species. Again, we could not test this second hypothesis directly. Indirectly, a count of the number of extreme specialists (those fungi with fewer than 20 interactions across all sampling times) between the two successional stages did not differ but the metrics of robustness suggested there was a greater extinction probability in the young successional stage.

Finally, the old growth forests at this site have been undisturbed for 130–140 years [18], [31] which may be long enough to allow for selection of specialization among plants and fungi at these sites [49], [50]. Recent research conducted at the older successional stage site demonstrated that very few specialist AM fungal species colonized a novel plant host's roots [51]. This research either supports the notion that specialist associations may take time to develop, or that early successional plants (such as invasives) may also favor generalist AM fungi (if doing so allows them to quickly obtain partners in a new environment) [52]. If selection for host specialization does not result in speciation and involves the same species present in the younger successional stage, it would be difficult to separate this hypothesis from the first hypothesis presented above. If the evolution of specialization involved speciation (perhaps a less likely scenario) then it would be difficult to distinguish between this hypothesis and the second hypothesis presented above. As result, there are several possible (non-mutually exclusive) explanations for the differences in the proportion of specialists between the two sites.

### Seasonal dynamics influenced network structure

AM fungal-plant network structure was also significantly altered by seasonal dynamics. As we predicted, the number of links between partners increased between the June and October samples. A similar pattern was observed in an arctic plant-pollinator network [7]. An increase in the number of associations between AM fungi and plants throughout a growing season has been shown in controlled greenhouse conditions (reviewed in [17]), however it has never before been demonstrated in field conditions. Previous research suggests that AM fungi differ in their colonization strategies [53] and may vary associations with host plants based on nutrient availability [54] which would contribute to variation in link density throughout the plant growing season if fewer links were maintained during the winter. This is also supported, as we predicted, by the increase in generality seen throughout the growing season where the relative number of plant species associated with a single AM fungal species increased from the first to the third sampling time. In addition, different AM fungal species are adapted to different temperature regimes [55], so fewer AM fungal species may be able to colonize roots during colder months but these same cold weather colonizers may also be able to maintain colonization at higher temperatures [56]. As a result, season-scale temporal variation is likely an important determinant for the structure of mutualistic networks [57].

Here we provide strong evidence of switching between partners by AM fungi and plants in a natural system. As already shown within this system, the same AM fungal species were observed across the growing season [58]. However, specific partnerships between plants and AM fungi were not consistent throughout the growing season, as supported by the high rate of turnover for links (on par with turnover rates between years [44]). In particular, the turnover rate for AM fungi was significantly greater than for plants—a somewhat surprising result given that AM fungi might be expected to be more stable partners due to their limited dispersal capabilities. Liu *et al.* [24] also showed variation in the presence and absence of different AM fungal phylotypes at different time points throughout the growing season. Switching among partners by AM fungi and plants suggests that AM fungal species and/or plant species have niches within the mutualism. That is, different partners may be better adapted or suited for different growth stages, soil temperatures [55], day lengths, or abiotic [17] or biotic stresses [21], [59]. When the abiotic or biotic environment changes the niche may disappear and plants and/or fungi may take on or adapt to a niche that requires different partners.

Switching between partners has consequences for our understanding of AM fungal-plant dynamics. The majority of studies focused on AM fungal-plant dynamics have been conducted under controlled conditions [17], assumed constant AM fungal communities in the roots throughout the experiment, and ignored variation in niches between the greenhouse and the field. As a result, we encourage new research identifying what aspects of temporal dynamics influence switching of partners between AM fungi and plants.

In contrast to our prediction there was no interaction between the effects of seasonal and successional dynamics on network properties. In particular, connectance was not magnified by both seasonal and successional dynamics.

### Conclusions

This network analysis has demonstrated several novel results. First, network metrics were generally consistent between replicate plots. This means that across spatial scales the effects we report here are conserved within treatment (succession) and across time, and because of this conservation can probably be extended to other AM fungal-plant systems, although more experiments at larger spatial scales are necessary. Second, succession can strongly influence mutualist network structure, primarily through a decrease in the number of interactions and an increase of the proportion of specialists within the community with increasing successional stage. Third, the AM fungal-plant mutualism structure is dynamic throughout the growing season as the number of links between species increases. Further, our analysis revealed that AM fungal and plant species partnerships change throughout the growing season, potentially reflecting shifts in biotic and abiotic conditions.
